# Applying the index of watershed integrity to the Matanuska-Susitna basin

**DOI:** 10.1080/15230430.2020.1800219

**Published:** 2020-08-27

**Authors:** Kelsey B. Aho, Joseph E. Flotemersch, Scott G. Leibowitz, Matthew A. LaCroix, Marc H. Weber

**Affiliations:** a Oak Ridge Institute for Science and Education (ORISE) Fellow C/o U.S. Environmental Protection Agency, Corvallis, Oregon, USA;; b Center for Environmental Measurement and Modeling, U.S. Environmental Protection Agency, Cincinnati, Ohio, USA;; c Center for Public Health and Environmental Assessment, U.S. Environmental Protection Agency, Corvallis, Oregon, USA;; d Region 10, Alaska Operations Office, U.S. Environmental Protection Agency, Anchorage, Alaska, USA

**Keywords:** Catchment integrity, cold climate hydrology, culvert remediation, sub-Arctic, watershed integrity

## Abstract

The Matanuska-Susitna Borough is the fastest growing region in the State of Alaska and is impacted by a number of human activities. We conducted a multiscale assessment of the stressors facing the borough by developing and mapping the Index of Watershed Integrity (IWI) and Index of Catchment Integrity (the latter considers stressors in areas surrounding individual stream segments exclusive of upstream areas). The assessment coincided with the borough’s stormwater management planning. We adapted the list of anthropogenic stressors used in the original conterminous United States IWI application to reflect the borough’s geography, human activity, and data availability. This analysis also represents an early application of the NHDPlus High Resolution geospatial framework and the first use of the framework in an IWI study. We also explored how remediation of one important stressor, culverts, could impact watershed integrity at the catchment and watershed scales. Overall, we found that the integrity scores for the Matanuska–Susitna basin were high compared to the conterminous United States. Low integrity scores did occur in the rapidly developing Wasilla–Palmer core area. We also found that culvert remediation had a larger proportional impact in catchments with fewer stressors.

## Introduction

The rivers of southcentral Alaska are essential to the fishery-related social, cultural, spiritual, and economic well-being of residents and visitors (Costanza et al. 1997; Colt and Schwörer 2009; Davis and Davis 2009). These human–fish interactions have been nourished for millennia by the regional aquatic and terrestrial landscape (Dynesius and Nilsson 1994; Holmlund and Hammer 1999; Ye, Yang, and Kane 2003; Brooks et al. 2012). Though the region’s mountain peaks and glaciers can seem pristine—especially when compared to the conterminous United States—the Matanuska-Susitna Borough faces an increasing number of deleterious impacts as the fastest growing region of the State of Alaska. The population doubled in the last thirty years (Matanuska–Susitna Borough 2015). To support the 100,000+ people, public and private infrastructure have similarly multiplied (Smith and Speed 2013).

This study analyzes the watershed integrity of the Matanuska-Susitna basin by way of human (i.e., anthropogenic) stressors such as infrastructure, stormwater pollution, and culverts. Anthropogenic stressors in a watershed can occur as discrete entities (e.g., culvert) or exist in a more continuous form (e.g., impervious land cover). They may be physically present in the stream itself (e.g., dams) or present on the adjacent landscape (e.g., agriculture; Roth, Allan, and Erickson 1996; D. Allan, Erickson, and Fray 1997; Harding et al. 1998). And though we generally think of these stressors in terms of how they impact the aquatic components, their presence can also impact adjacent marine (Robards et al. 1999; Stroeve et al. 2012), atmospheric (Shindell and Faluvegi 2009), and terrestrial (Jia, Epstein, and Walker 2003; Schuur et al. 2015) components of the watershed.

To track and better understand how the rapid growth was impacting streams in the Matanuska-Susitna basin, two studies have analyzed the human disturbances at the ten and twelve-digit hydrologic unit (HU) scale: *Salmon Watersheds in the Mat-Su Basin: A Map Atlas to Prioritize Conservation* (Smith 2009) and the *Human Disturbance in the Mat-Su* study conducted in association with the National Fish Habitat Action Plan (Esselman et al. 2011). However, cumulative impacts were not calculated or included in either of these studies. Note that HUs delineate the boundaries of surface water drainage units. HUs have been delineated at six scales. The smallest scale HUs (i.e., subwatersheds) often only represent part of a drainage network such that multiple HUs are required to define an entire drainage area of an outlet point. Studies using HUs, such as these two studies, typically combine data within the HUs but do not accumulate the data downstream using stream network information.

A local and downstream understanding of watershed structure and function is critical for successful management of these landscapes (Thorp et al. 2013). Landscape-scale (e.g., watershed-scale) assessments are less common than site- or reach-scale studies due partially to their need for data from multiple ecosystems (e.g., aquatic and terrestrial) and management authorities. In addition to visualizing the combination of stress locally and the accumulation downstream, watershed-scale analyses can also be used to evaluate terrestrial stressors such as agriculture. To address this need for downstream accumulation, Flotemersch et al. (2016) developed the Index of Watershed Integrity (IWI). The IWI is an aggregate index ranging from zero (low integrity) to one (high integrity) based on first-order approximations of relationships between stressors and six key watershed functions: hydrologic regulation (HYD), regulation of water chemistry (CHEM), sediment regulation (SED), hydrologic connectivity (CONN), temperature regulation (TEMP), and habitat provision (HABT).

To date, the IWI has been applied to the conterminous United States (Kuhn et al. 2018; Thornbrugh et al. 2018; Johnson, Leibowitz, and Hill 2019) and to four basins in the western Balkans (Aho et al. 2020). In these applications, the IWI was built on data available for the study area to provide a consistent means of examining watershed integrity across the entire area. Combined, these studies and Scown et al. (2017) demonstrate how the Index of Catchment Integrity (ICI) and IWI can be useful management tools at multiple scales, especially when used with information from other sources of big data.

In the present study, our first objective was to adapt the IWI to the human activities in the Matanuska-Susitna basin region. Incorporation of regionally relevant stressors would allow for consideration of stressors critical to, and actionable by, watershed managers of the study area. Our second objective was to demonstrate how information provided by the IWI could be used to create a scenario of value to decision makers and environmental planners.

## Methodology

### Study area

**Figure 1.**
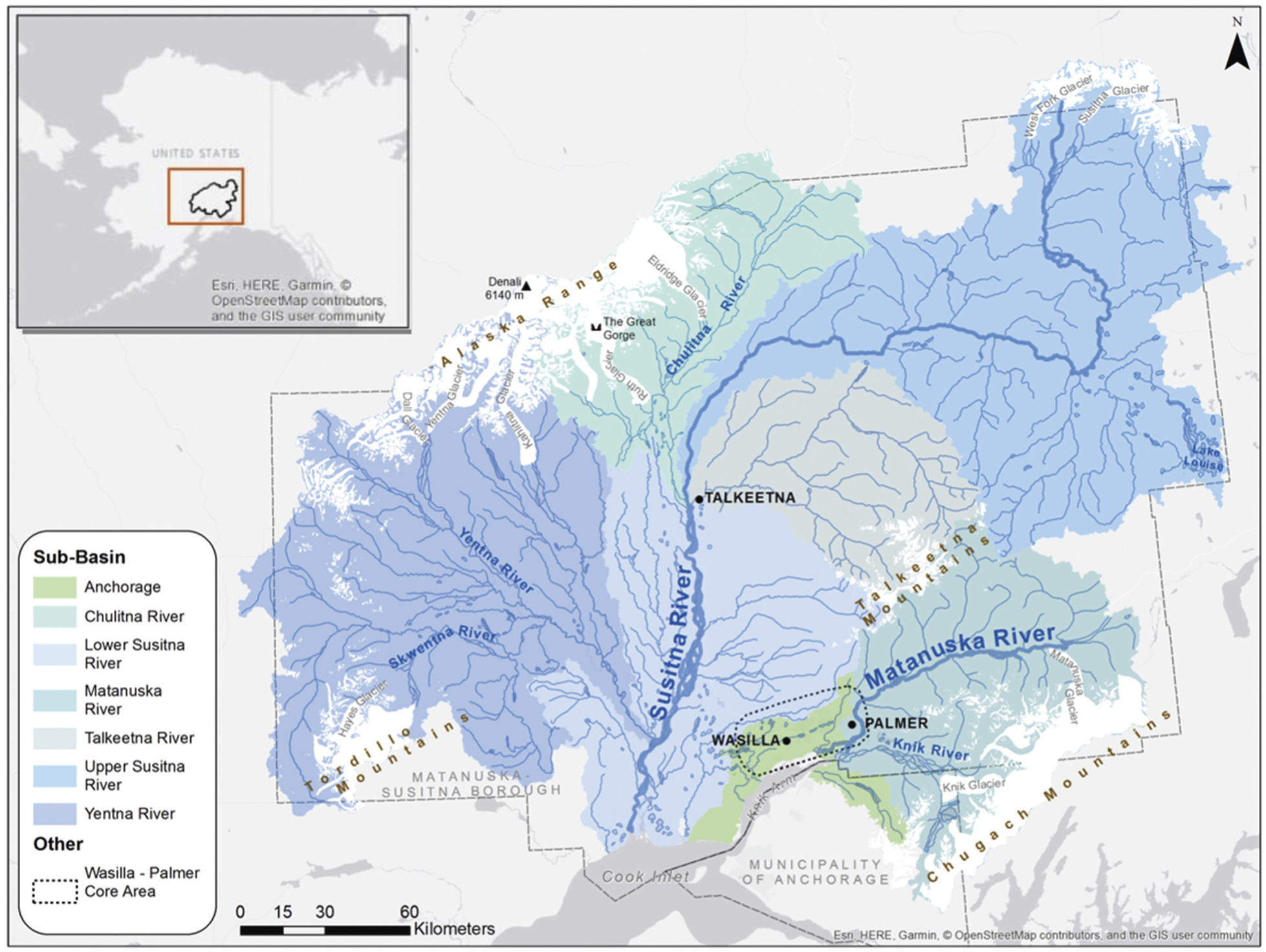
Map of the Matanuska-Susitna basin.

This study was conducted on the Matanuska-Susitna basin located in southcentral Alaska. Though referred to as a single basin, the Matanuska-Susitna basin is a combination of the Matanuska River watershed, Susitna River watershed, Knik River watershed, and several smaller rivers that drain to upper Cook Inlet. As a management unit, the Matanuska-Susitna basin consists of seven eight-digit HUs encompassing a combined area of 65,024 km^2^ (Figure 1). The area is two magnitudes smaller than the conterminous United States IWI application (8,000,000 km^2^; Thornbrugh et al. 2018). The hydrologic region of the Matanuska-Susitna basin is characterized by glacially derived valleys bounded by the Alaska Range to the north and the Talkeetna and Chugach Mountains to the east (Callegary et al. 2013). From the mountains, the Matanuska River and the Susitna River both drain to upper Cook Inlet south of the Matanuska-Susitna valley. There they deposit 20 million tons of sediment annually (Mulherin et al. 2001). As part of the Cook Inlet ecoregion (#115; Gallant et al. 1995), the low elevations of the Matanuska-Susitna basin are generally free of permafrost and home to most of the residents of Alaska. At higher elevations are icefields, glaciers, and few permanent human settlements, as well as many extractable resources (e.g., gold, silver, lead, copper, coal, uranium, antimony; Gallant et al. 1995).

The Susitna River originates in the Upper Susitna subbasin (i.e., eight-digit HUs), at the terminus of the Susitna and West Fork Glaciers. After flowing along the southeastern face of the Alaska Range, the Susitna River turns south through the Lower Susitna River subbasin before reaching Cook Inlet, west of Anchorage. The Yentna, Chulitna, and Talkeetna Rivers contribute to the Susitna River. The Skwentna River begins at Hayes Glacier in the Tordrillo Mountains before joining with the Yentna River, which flows from the terminus of the Yentna and Dall Glaciers. Runoff from the largest glacier in the study area, the Kahiltna, also feeds the Yentna River. The source of the Chulitna River is the Eldridge Glacier. The Chulitna River is characterized by Denali, the highest point in North America at 6,140 m, and the Ruth Glacier, which has carved the 2,743-m-deep Great Gorge, the deepest gorge in the world (as comparison, the Grand Canyon is 1,857 m deep; Wilford 1992). The well-known white water of the Talkeetna River flows from the Talkeetna Mountains before joining the Susitna River just south of the town of Talkeetna. Cumulatively, the river and stream reaches in the Susitna River basin have a drainage area of 53,099 km^2^ (NHDPlus HR 2018). The winter period, when river surfaces are frozen, begins in early November and ends in May when the ice breaks up (Mulherin et al. 2001). Nearly all of the ice present in Cook Inlet is contributed by the Susitna River, which has the fifth largest discharge rate (United States Geological Survey [USGS] 1990), of 1,400 m^3^/s (USGS Gauge 15294350; USGS 2020), in Alaska.

The Matanuska River flows west from the Matanuska Glacier, bound by the Chugach Mountains to the south and Talkeetna Mountains to the north. Immediately after meandering through Palmer, Alaska, the Matanuska River joins with its largest tributary, the silty Knik River, which originates from Knik Glacier. The combined rivers then flow into Knik Arm of Cook Inlet. The Matanuska River drainage area is 8,720 km^2^ (NHDPlus HR 2018) and has an average annual discharge rate of 115 m^3^/s (USGS Gauge 15284000; USGS 2020). The fraction of the northern Anchorage eight-digit HU was included as part of the study area because it is managed as part of the Matanuska-Susitna basin. This drainage area is 1,511 km^2^. Other parts of the Anchorage eight-digit HU drain directly to Cook Inlet and are not managed as part of the Matanuska-Susitna basin and therefore are not included in our analysis.

### Geospatial framework

The IWI uses the framework and terminology of the National Hydrography Dataset Plus Version 2 (McKay et al. 2012; NHDPlusV2 2017). “From-to” relationships have been applied to stream segments to derive cumulative watershed metrics. The geospatial framework of the IWI is hierarchically organized starting with streams and their riparian zones embedded in local catchments. Each streamline (or in select cases multiple ephemeral streamlines or multiple features such as canals/ditches or side channels) corresponds with a single, unique catchment (NHDPlus HR 2018).

It is important to clarify that the terms “catchment” and “watershed” have specific meanings in the NHDPlusV2 framework and, consequently, the IWI. A catchment is defined as the area of a landscape (i.e., local drainage area) that contributes flow directly to a stream segment, excluding upstream contributions (catchments can also lack a stream segment in certain instances; i.e., so-called sink catchments; McKay et al. 2012). The watershed of that stream segment includes the catchment plus all upstream catchments. We created riparian zones in the same manner as used in Thornbrugh et al. (2018), defining riparian zones as a 100-m buffer on either side of the streamline. At the time of writing this article, the only National Hydrography Dataset available for the Matanuska-Susitna basin was the NHDPlus High Resolution (NHDPlus HR). Compared to NHDPlus and NHDPlusV2, which were not available for the Matanuska-Susitna basin, the NHDPlus HR data add an order of magnitude of detail to streamlines and catchments. This study therefore pilots the use of NHDPlus HR. This piloted use of NHDPlus HR will be relevant for the future applications of the IWI to the entire State of Alaska and the conterminous United States.

Average size of catchments depends on the resolution of the hydrographic data layer available for a study area. For example, in the conterminous United States, the average NHDPlusV2 catchment was 3.11 km^2^ (Thornbrugh et al. 2018), whereas in a study conducted in the western Balkans (Aho et al. 2020), the average catchment was 36.4 km^2^ (European Environment Agency 2012). In the current study, using the NHDPlus HR, the mean surface area of Matanuska-Susitna basin catchments is 0.38 km^2^. For the most upstream catchment in a system, the area of the catchment and watershed is the same because there are no additional upstream catchments. Proceeding downstream, the area of watersheds progressively increases with the largest watershed being associated with the most downstream catchment of the system. Catchments, on the other hand, need not progressively increase in size, given that they only represent the landscape area of a specific stream segment.

### Data acquisition

Previous applications of the IWI to the conterminous United States (e.g., Thornbrugh et al. 2018; Johnson, Leibowitz, and Hill 2019) were based on the list of major stressors identified by Flotemersch et al. (2016) for the six key functions of the IWI. This candidate list of data layers was refined by Thornbrugh et al. (2018) when watershed integrity was first mapped for the conterminous United States. In the application of the IWI to the western Balkans (Aho et al. 2020), the same data layers were not available, requiring the identification of surrogates where possible. Criteria used for the selection of these surrogate data layers included the following:

- Comparable purpose with data sets used for the conterminous United States IWI
- Spatial coverage of the entire study area
- Public availability
- Peer review.

To adapt the IWI to the stressors and geography of the Matanuska-Susitna basin, and given the objective of adapting the IWI to the regionally relevant stressors published by the partnership, a slight modification of the data layer criteria was required. Specifically, the first criterion was modified to consider a number of factors articulated in the partnership’s action plan and by Smith (2009) and Smith and Speed (2013) in their analysis of potential threats to salmon and salmon habitat: road density; culverts that impede fish passage; converted and impervious land cover; platted subdivisions; water quality; invasive northern pike; conservation management status; instream flow reservations; aquatic invasive species; climate change; development in estuaries and nearshore habitats; ground and surface water withdrawals; household septic systems and wastewater; large-scale resource development; motorized off-road recreation; residential, commercial, and industrial development; roads and railroads; and stormwater runoff.

Our review of the above factors resulted in a list of eighteen stressors that were associated with the six key functions and for which data were available for the Matanuska-Susitna basin (Table 1). Several of these were aggregate stressors; for example, density of transportation included density of airports, roads, railroads, and trails. Of the primary stressors, fourteen were acquired specifically for this Matanuska-Susitna basin application (see Table S1 for details on methods of calculation and data sources).

To calculate ICI and IWI, statistics of the individual stressors listed in Table 1 were calculated based on the boundaries of the 166,778 catchments in the study area. For example, the density of subdivision perimeters within each of the catchments were calculated for density of cadastral subdivisions (see Table S1 for the calculations used for each stressor). However, Thornbrugh et al. (2018) found that there could be high correlation between stressors (e.g., percentage of agricultural land cover and K-factor soil erodibility). Thornbrugh et al. (2018) removed a single stressor if there were two stressors of a single functional component with correlation >0.7. Such a correlation between two variables could denote that the variables were indicators of the same fundamental stressor, leading to double counting and giving the stressor too much weight. To address this double counting concern, we calculated correlations between nonaccumulated catchment stressors and between accumulated watershed stressors. We found one ICI correlation >0.70 (density of septic and sewer and density of cadastral subdivisions) and two IWI correlations >0.70 (density of contaminated sites and density of septic and sewer; density of septic and sewer and density of cadastral subdivisions). Because the correlations did not occur between stressors within the same functions, this did not represent double counting and the stressors were not dropped (including correlated stressors in different functions was not considered double counting because these could be unrelated mechanistically).

(1) $${\hat{ICI}}_{i,c}=\frac{\sum_{j=1}^{n_{i,c}}\left[1-\left(\frac{s_{j,c}}{s_{j,\text{cmax}}}\right)\right]}{n_{i,c}}$$

We then used these stressor statistics to calculate the ICI based on the revised definition by Aho et al. (2020), where ICI_*i,c*_ is the ICI estimator of the *i*th functional component for catchment *c, s*_*j,c*_ is the value for the *j*th stressor in catchment *c, s*_*j,c*max_ is the maximum (worst attainable) value for the *j*th stressor across all catchments, and *n*_*i,c*_ is the number of stressors associated with the *i*th functional component that could possibly occur in catchment *c*. ICI scores are useful for characterizing and monitoring the extent of local stressors. ICI scores are also essential for accumulating impacts, which we refer to as the IWI. Table S2 contains *s*_*j,c*max_ values for each of the eighteen stressors, along with several other stressor characteristics at the catchment scale.

(2) $${\hat{IWI}}_{i,w}=\frac{\sum_{j=1}^{n_{i,w}}\left[1-\left(\frac{s_{j,w}}{s_{j,\text{cmax}}}\right)\right]}{n_{i,w}}$$

We used the accumulated (watershed) stressor values to calculate the IWI, which is based on Flotemersch et al. (2016) and Thornbrugh et al. (2018) and defined by Aho et al. (2020) as where ${\hat{IWI}}_{i,w}$ is the IWI estimator for the *i*th functional component for watershed *w, s*_*j,w*_ is the value for the *j*th stressor in watershed *w, n*_*i,w*_ is the number of stressors associated with the *i*th functional component in watershed *w*, and *s*_*j,c*max_ is the maximum value for the *j*th stressor across all watersheds. Note that both *n*_*i,c*_ and *n*_*i,w*_ are based on the types of stressors of concern that could potentially occur in a catchment or watershed and not on the actual presence of the stressor. For example, *n*_*i,c*_ will not be decreased by one for a catchment that does not have culverts, because culverts could occur in that catchment. However, *n*_*i,c*_ is decreased by one if a stressor of concern cannot occur in a particular catchment; for example, density of roads in catchment riparian areas cannot be defined in sink catchments that have no stream segments and so cannot have any associated riparian area (McKay et al. 2012; Thornbrugh et al. 2018). As another example, impervious surface data only exist for about half of the Matanuska-Susitna basin study area, and so *n*_*i,c*_ was decreased by one for catchments where impervious surface data were not available.

To discern spatial trends, we analyzed pairwise correlations between each of the six functional components. Using ICI and IWI results, we calculated means of each based on the subwatersheds and compared the results with scores in the Wasilla-Palmer core area.

### Evaluation of ICI and IWI indices

To evaluate the Matanuska-Susitna basin ICI and IWI results, we compared our results with two previous analyses of anthropogenic stressors in the Matanuska-Susitna basin: *A Map Atlas to Prioritize Conservation* (Smith 2009) and *Human Disturbance in the Mat-Su*, conducted in association with the National Fish Habitat Action Plan (Esselman et al. 2011).

The *Map Atlas to Prioritize Conservation* (Smith 2009) considered road density, culverts, impervious and converted land, platted subdivisions, water quality, invasive pike, conservation management status, and instream flow reservations based on the statewide portfolio of areas of biological significance (Feirer, Couvillion, and Smith 2006) and used the USGS’s tenand twelve-digit HUs for its geospatial framework (Seaber, Kapinos, and Knapp 1987). The *Human Disturbance in the Mat-Su* considered fourteen human disturbance variables in Alaska and provided information on levels of disturbance at twelve-digit HUs. Results showed that low levels of anthropogenic disturbance are present in many areas of the state, with higher values present in the urban areas around Anchorage, Fairbanks, and Juneau as well as along the main transportation corridors.

Pearson correlation coefficients (*r*) between ICI and IWI results and the Matanuska-Susitna basin-specific map atlas (Smith 2009) and the nonaccumulated data from the statewide fish habitat disturbance (Esselman et al. 2011) were calculated. Smith (2009) and Esselman et al. (2011) summarized human disturbances in tenand twelve-digit HUs to create a combined disturbance index, as proposed by Danz et al. (2007). To statistically compare our results with Smith (2009), we calculated the spatially weighted mean ICI and accumulated IWI of catchments in each of the 329 ten- and twelve-digit HUs identified for the V1 vulnerability map. Using the same methodology, we compared our results with the 596 twelve-digit HUs used by Esselman et al. (2011).

### Culvert scenario

In partnership with stakeholders in the Matanuska-Susitna basin, we selected density of culverts for scenario development. Culverts can be a significant stressor to salmon, a source of cultural and economic importance in the Matanuska-Susitna basin. The intended purpose of the scenario was to evaluate how culvert remediation could improve the local and downstream integrity. We built the scenario on the Matanuska-Susitna basin ICI and IWI data and a culvert data set (Alaska Department of Fish and Game [ADFG] 2019) of 558 barriers in 430 catchments in the Matanuska-Susitna basin (Eisenman and O’Doherty 2014). The ADFG data set attributed an *R* factor to individual culverts based on likelihood of impeding fish passage (Mark Eisenman, pers. comm.). An *R* value of 1 was assigned to culverts likely to impact fish passage, 0.5 to culverts that may impact fish passage, and 0 to culverts likely representing no impact to fish passage. Culverts for which survey data were not available were given a 0 with full acknowledgment that they may indeed represent a barrier. Culverts that have a downstream drop or “perch” ≥0.3 m (i.e., 1 ft), a barrier to juvenile salmonids, were attributed an additional 0.5, thus resulting in a max *R* factor value of 1.5 for any individual culvert.

Using the existing culvert data set, the scenario was as follows: If one or more culverts that “likely impact fish passage” or “may impact fish passage” are in a catchment or watershed, the culvert with the worst *R* factor (greatest value) is improved to “no impact on fish passage” or a value of 0.

The scenario hypothesized remediation of one culvert per catchment (where one or more existed). Though the scenario automatically decreased the number of culverts requiring remediation in the catchment and watershed by one, the maximum number of culverts (*s*_*j;c*max_) in the catchment or watershed was unchanged. The scenario was iteratively run until all culverts were remediated. Each iteration removed the culvert with the worst *R* factor. Upon removal of each culvert, the ICI and IWI values associated with that catchment and watershed were recalculated to provide the change in catchment and watershed integrity associated with each culvert remediation. Though remediating a culvert would normally be evaluated in terms of its benefit in improving fish passage, calculating the changes in ICI and IWI would allow an environmental manager to consider the effects of these changes at larger catchment and watershed scales. This would also allow managers to visualize where culvert remediation would have the greatest benefit with respect to catchment and watershed integrity (i.e., which catchments and watersheds had the greatest changes in ICI and IWI, respectively).

## Results

The following results range from 0 (low integrity) to 1 (high integrity).

### Index of catchment integrity

**Figure 2.**
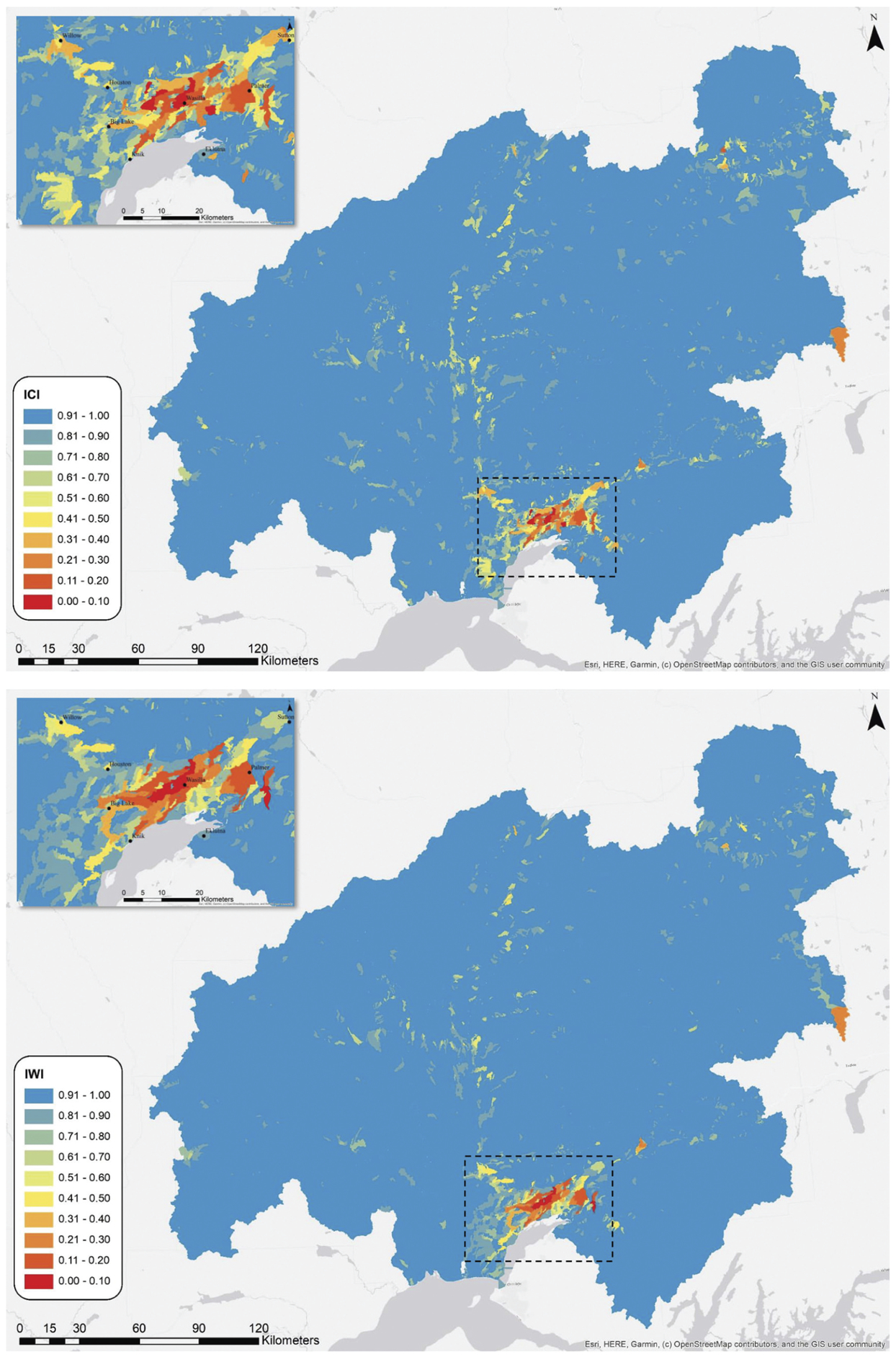
Maps of the ICI results (top) and the IWI results (bottom).

The mean ICI of the Matanuska-Susitna basin (Figure 2) was 0.995 (SD = 0.031). Individual catchment ICI values ranged from 0.03 to 1.00 with a median value of 1.00. In comparison, minimum, mean, and maximum ICI values for the conterminous United States were 0.02, 0.61, and 0.95, respectively (Johnson, Leibowitz, and Hill 2019). The mean catchment functional component values for the Matanuska-Susitna basin were 0.998 (HYD; SD = 0.007), 0.999 (CHEM; SD = 0.008), 0.999 (SED; SD = 0.011), 0.999 (CONN; SD = 0.008), 1.000 (TEMP; SD = 0.008), and 0.998 (HABT; SD = 0.014).

The mean ICI values of the Matanuska-Susitna basin subbasins (i.e., eight-digit HUs) were 0.935 (Anchorage; SD = 0.151), 0.998 (Chulitna; SD = 0.023), 0.989 (Lower Susitna; SD = 0.044), 0.997 (Matanuska; SD = 0.025), 0.997 (Talkeetna; SD = 0.017), 0.995 (Upper Susitna; SD = 0.031), and 0.998 (Yentna; SD = 0.016). A large majority of the catchments with ICI values below 0.90 were in the Wasilla-Palmer core area (Figure 2).

### Index of watershed integrity

The mean IWI of the Matanuska-Susitna basin was 0.993 (SD = 0.039). Individual watershed IWI values ranged from 0.03 to 1.00 with a median value of 1.00. Minimum, mean, and maximum for the conterminous United States IWI were 0.06, 0.58, and 0.95, respectively (Johnson, Leibowitz, and Hill 2019). The mean watershed functional components values for the Matanuska-Susitna basin were 1.000 (HYD; SD = 0.006), 0.999 (CHEM; SD = 0.011), 0.998 (SED; SD = 0.014), 0.999 (CONN; SD = 0.011), 0.999 (TEMP; SD = 0.013), and 0.998 (HABT; SD = 0.014).

The mean IWI values of the Matanuska-Susitna basin subbasins (i.e., eight-digit HUs) were 0.876 (Anchorage; SD = 0.231), 0.997 (Chulitna; SD = 0.021), 0.983 (Lower Susitna; SD = 0.042), 0.996 (Matanuska; SD = 0.030), 0.997 (Talkeetna; SD = 0.016), 0.992 (Upper Susitna; SD = 0.029), and 0.998 (Yentna; SD = 0.014). A majority of the watersheds with IWI values below 0.90 were in the Wasilla-Palmer core area (Figure 2).

### Evaluation of the ICI and IWI

The Matanuska-Susitna basin ICI and IWI results were correlated at *r* = 0.5759. Correlations between ICI and IWI are expected; for example, Thornbrugh et al. (2018) found a correlation of 0.85 between the conterminous United States IWI and ICI. They found this was mostly due to high correlations (*r* > 0.9) within small watersheds (areas of 10 km^2^ or less), compared to low correlations (*r* < 0.5) within larger watersheds (areas of 10,000 km^2^ or more). Note that catchments in small watersheds have higher spatial autocorrelation than those in large watersheds. However, the current analysis makes use of NHDPlus HR catchments, and it is therefore possible that the scale at which these high spatial autocorrelations occur is reduced. This matter requires further investigation.

To facilitate the comparison of the higher-resolution ICI and IWI values for the 166,778 NHDPlus HR catchments and watersheds of the Matanuska-Susitna basin, respectively, results were aggregated to lower-resolution levels of the 329 ten-digit HUs and 596 twelve-digit HUs in Smith (2009) and the 596 twelve-digit HUs in Esselman et al. (2011). The mean vulnerability (V1) (Smith 2009) value was 2.131 (SD = 0.704) and ranged from 0.15 (best) to 6.55 (worst). The mean human disturbance (Esselman et al. 2011) value was 0.096 (SD = 0.150) and ranged from 0 (best) to 1 (worst). Correlations (*r*) of the aggregated ICI and the IWI with the vulnerability (V1) of the lower Matanuska-Susitna basin (Smith 2009) were −0.633 and −0.631, respectively. Correlations (*r*) of the aggregated ICI and IWI values and human disturbances (Esselman et al. 2011) were −0.755 and −0.787, respectively.

### Culvert scenario

In the Matanuska-Susitna basin, the maximum number of culverts documented by ADFG in a catchment was 10. The *R* factor sum of the 558 culverts was 318. The maximum cumulative *R* factor in a single catchment was 6.5.

**Figure 3.**
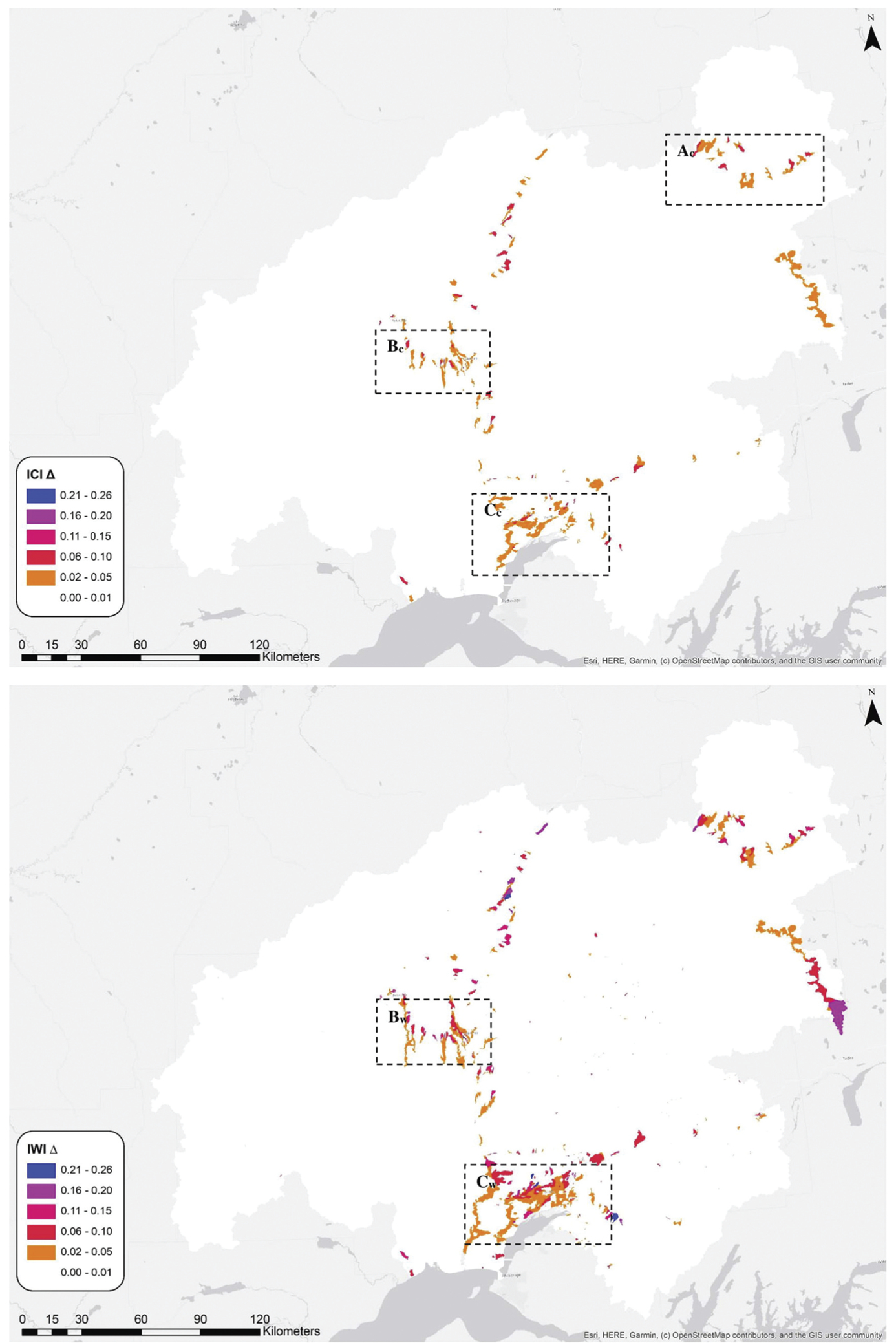
Map of the changes to ICI values caused by scenario 1 (top) and map of the changes to IWI values caused by scenario 1 (bottom) where *A*_*c*_ highlights large changes to ICI scores in catchments with high integrity along the Denali Highway; *B*_*c*_ highlights large changes to ICI scores in catchments with high integrity along the Petersville Road, and *B*_*W*_ highlights large downstream changes to IWI scores in watersheds with high integrity; *C*_*c*_ highlights the relatively small changes to ICI scores in the highly developed core area compared to *C*_*W*_, which highlights larger changes to the IWI scores or the accumulated downstream impact of scenario 1.

Under our scenario, the mean change to ICI or IWI scores upon remediation of the present culverts was 0.0004 (SD = 0.0025) and 0.0005 (SD = 0.0058), respectively. The largest increase to the value of an individual catchment was 0.1678 and the largest increase to an individual watershed was 0.2647 (see Figure 3). The largest increase to a mean catchment functional component was 0.0005 (CHYD) and the largest increase to a mean watershed functional component was 0.0002 (WCONN). The largest increase to a minimum value of catchment functional component was 0.0706 (CHYD) and the largest increase to a watershed functional component was 0.0225 (WCONN).

## Discussion

The ICI and IWI are tools for determining the integrity of catchments and watersheds; that is, the extent of disturbance by anthropogenic stressors. The degree of integrity can be related to the capacity of a catchment or watershed to provision ecosystem services. The tools rely heavily on satellite- and permit-derived data to calculate the integrity of six key functions of catchments and watersheds (Flotemersch et al. 2016; Thornbrugh et al. 2018). Here we explored how the ICI and IWI can be used to better support regional decision making. Specifically, we adapted the ICI and IWI to the geography, unique stressors, and data availability of the Matanuska-Susitna basin in southcentral Alaska. Via partnership with those working in the basin, we explored local data on stressors relevant to this study area. This Matanuska-Susitna basin application also used newly available NHDPlus HR data, which offers higher resolution than the NHDPlusV2 data used on previous conterminous United States ICI and IWI applications (NHDPlus HR 2018).

In comparison to the conterminous United States ICI and IWI, integrity scores of the Matanuska-Susitna basin were generally high, indicating lower stress levels in the basin. Areas incurring the greatest stress were in the rapidly developing Wasilla-Palmer core area (Figure 2). In this area, the analysis showed notable impairment to sediment regulation, temperature regulation, and habitat provision. These findings are consistent with what would be expected in an area with rapid population growth (Wang et al. 2001; Nelson and Palmer 2007; Wickham et al. 2014).

Availability of NHDPlus HR data resulted in use of catchments that are an order of magnitude smaller than the NHDPlusV2 catchments used for the conterminous United States IWI application. This is significant because it resulted in ICI and IWI data at the local or even household scale. Availability of ICI and IWI data at a catchment scale averaging 0.3 km^2^ provides information, easily perceived, to resource managers. It is also a scale conducive to outreach activities with private landowners, communities, and industry and so makes results more tangible to those groups. And as pointed out by Van Jaarsveld et al. (2005) and Bennett, Peterson, and Gordon (2009), management of multiscale ecosystem services should likewise be implemented at multiple scales.

One such multiscale stressor is culverts. We piloted a scenario that provided a tool for local (i.e., catchment-scale) and regional (i.e., watershed-scale) planning. In this remote region, culvert remediation was a practical scenario to pilot because most culverts are located along the road system, making them relatively accessible. A scenario based on mine or timber harvest could have been discounted by the inaccessibility (and high transport cost) of such a prescribed action. The scenario results documented how the ICI and IWI scores change in response to culvert remediation. In catchments with few stressors, culvert remediation had a larger proportional impact on the ICI and IWI scores than in catchments with more stressors. For example, the ICI scores for catchments along the largely unpaved and unpopulated Denali Highway had some of the largest increases (Figure 3: *A*_*c*_). Culvert remediation in watersheds with relatively high integrity also impacted the IWI scores downstream from the culvert (compare the Petersville Road in Figure 3: *B*_*c*_ and *B*_*w*_). For developed areas with high concentrations of culverts and other stressors, we found that the impact of remediation on the catchment ICI scores (Figure 3: *C*_*c*_) was negligible compared with the change to the IWI scores downstream (Figure 3: *C*_*w*_). As a decision support tool, the culvert remediation scenario could be used in combination with other information on the site-specific impact on salmon to prioritize remediation. The scenario could be used to predict potential impacts of new culverts, for example, by adding culverts to catchments with road development plans. Such a decision support tool could aid citizens, planners, regulators, and agencies, working at various spatial scales, in visualizing potential impacts of future development.

Scenarios could further be used to support environmental impact assessments and compensatory mitigation discussions. Compensatory mitigation under Section 404 of the Clean Water Act (U.S. Congress 1972) refers restoration (reestablishment or rehabilitation), establishment (creation), enhancement, and/or in certain circumstances preservation of wetlands, streams, and other aquatic resources for the purposes of offsetting unavoidable adverse impacts that remain after all appropriate and practicable avoidance and minimization have been achieved (Processing of Department of the Army Permits, 33 C.F.R. § 325 (1986); Compensatory Mitigation for Losses of Aquatic Resources, 33 C.F.R. § 332 (2008); Guidelines for Specification of Disposal Sites for Dredged or Fill Material, 40 C.F.R. § 230 (1980)). As an example, assume that an environmental impact assessment of a proposed development shows that a necessary culvert will have unavoidable adverse impacts to fish passage. In the context of the IWI, this would represent an impairment to hydrologic connectivity and potentially other functions. Alternative scenarios created using ICI and IWI data, along with other available data, could be used to explore development options that might minimize impairment to watershed functions. Scenarios could also be used to explore other options for compensatory mitigation in the basin to offset impacts of planned development.

We compared our results with studies by Smith (2009) and Esselman et al. (2011). These two studies estimated greater human disturbance and vulnerability, respectively, than the ICI and IWI results aggregated to ten- and twelve-digit HUs even though the ICI and IWI included more individual stressor data sets. One possible reason for this disparity is differences in data resolution. Specifically, it is possible that the lower resolution HUs used in the Smith (2009) and Esselman et al. (2011) studies led to an overestimation of the stressors. For example, consider road density. Esselman et al. (2011) identified 147 out of 519 twelve-digit HUs, or a total of 16,958 km^2^, with a road density >0. The ICI identified 6,798 out of 166,778 catchments, or a total of 8,118 km^2^, with a road density >0. Though evaluating a greater length of roads, the latter demonstrated less than half the area of Esselman et al.’s (2011) estimated impact by road density. Similar high estimations related to the resolution of the ten- and twelve-digit HU geospatial framework are thought to have comparatively inflated values of stressors based on vector data. The use of an NHDPlus HR geospatial framework may provide greater accuracy. Other factors that could cause differences between our results and those of the previous studies are differences in how the indices were calculated (the ICI and IWI combine the stressors and multiply the functions, whereas the other two studies only combine stressor), possible changes over time, and measurement of different stressors.

Additional differences among the approaches are the HUs used by Smith (2009) and Esselman et al. (2011), which were not accumulated as true watersheds and therefore did not allow for the consideration of cumulative impacts of stressors. Consequently, the Matanuska-Susitna basin ICI and IWI application provides information on more watershed stressors at a finer scale.

We acknowledge that the results of this IWI analysis were not validated with aquatic data as has been done for the conterminous United States IWI analysis (Kuhn et al. 2018; Thornbrugh et al. 2018; Johnson, Leibowitz, and Hill 2019). Though data do exist for the Matanuska-Susitna basin, these are generally collected opportunistically for project-specific purposes and mostly focus on the Wasilla-Palmer core area. There is a general lack of comprehensive data for the whole basin that would be required to fully validate our current analysis. However, there are likely certain areas with high variation in ICI/IWI scores that do have data, such as road-accessible streams. Data in such areas could provide weight of evidence for our hypothesized factors in future analyses. Further, we reiterate that ICI and IWI scores are first-order approximations of relationships between stressors and the identified watershed functions (i.e., use of negative linear relationships and no weighting; see Flotemersch et al. 2016; Johnson, Leibowitz, and Hill 2019). Paraphrasing Reichert and Omlin (1997), models drastically simplify complex ecological systems. This predicament was summed up by Chatfield (1995, 428) as “all models are wrong, but some are useful.” While acknowledging these limitations, we believe that there are opportunities for further research and that the ICI and IWI models could prove useful in facilitating communication between stakeholders, managers, and other invested entities on downstream impacts.

A limitation of our approach is that ICI and IWI values can only be calculated if catchment boundaries and stream network data are available. At the time of this study, the NHDPlusV2, which provides the ICI and IWI catchment geospatial framework essential for accumulating stressors, currently only exists for a few regions of the state. Though it is unlikely the NHDPlus HR will exist for the entire state in the next few years, regions often have access to the relevant stressor data. If there is an interest in conducting additional analyses in Alaska, perhaps the most practical approach would be to develop ICI and IWI analyses region by region as NHDPlus HR is made available.

An additional limitation of the ICI and IWI and similarly derived applications is that they rely on remotely derived or geographic information system data layers that are recaptured or updated according to different schedules. For example, the National Land Cover Dataset is released every five years for the conterminous United States (Wickham et al. 2014) and less frequently for Alaska. The 2016 National Land Cover Dataset was recently released for Alaska in April 2020 (after this analysis was completed). Yet, these older data are still valuable for tracking trends and identifying emerging issues. As new data become available, the Matanuska-Susitna basin ICI and IWI can be updated and used to facilitate trend analysis and improve detection of emerging risk factors such as climate change (e.g., glacial mass balance change: Beamer et al. 2016, 2017; deepening of the active layer of permafrost: Panda, Marchenko, and Romanovsky 2014).

While working on the adaptation of the IWI to Alaska, local colleagues drew attention to the fact that the IWI only accumulates stressors that flow downstream. The concept of upstream flow (e.g., salmonids transporting nutrients and energy upstream from the ocean; Schindler et al. 2003) and protective factors (e.g., an in-stream flow reservation) could both be topics of further research.

Future research geared toward improving the IWI modeling approach nationally should focus on the development of methods for weighting data sets (and resulting functional component subindices) based on the geomorphologic setting (J. D. Allan 2004) and weighting of the impact of stressors on IWI scores as a function of distance and hydrologic connectivity. Regarding the latter, in the current approach all ICI values for catchments draining to a given pour point are given equal weight. However, the effects of stressors vary with increasing distance (e.g., King et al. 2005; Peterson et al. 2011). Tools that consider these spatial relationships are becoming increasingly available and could contribute to improvement of the IWI approach (Peterson and Pearse 2017).

To maximize utility of this tool, we recommend that publicly available results be hosted on a user-friendly website, similar to the Kenai Decision Support Tool (Audubon Alaska 2018).

## Conclusion

In the present study, we demonstrated how the ICI and IWI can be applied at a regional scale by incorporating place-based anthropogenic stressors critical to, and actionable by, watershed managers of the immediate study area. More specifically, the Matanuska-Susitna basin ICI and IWI data were used to create a simple scenario examining the impact of culvert remediation on local ICI scores, accumulated IWI scores, and functional components. At the catchment scale, the largest improvements were to HYD scores, and at the watershed scale, the largest improvements were to CONN scores. Though these results may be intuitive, the example shows the power of the tool for adaptation planning and compensatory migration.

## Supplementary Material

Supplement1

Supplement2

## Figures and Tables

**Table 1.** Functional Components and Stressors of the Index of Catchment Integrity (ICI) and Index of Watershed Integrity (IWI). The stressors used for the ICI are accumulated at the catchment (CM) scale while the stressors used for the IWI are accumulated at the watershed (WS) scale. A ‘+’ denotes a stressor is included in a functional component, while a blank signifies it is absent.

		Functional Components					
		Hydrologic Regulation	Regulation of Water Chemistry	Sediment Regulation	Hydrologic Connectivity	Temperature Regulation	Habitat Provision
**Stressors**	DenCDP	+			+		
	DenCulv	+	+	+	+		
	DenHouseRp						+
	DenMat		+	+			
	DenMine		+	+			
	DenPoll		+			+	
	DenResvr	+	+	+	+	+	+
	DenSepSew		+		+	+	
	DenSubd				+		
	DenTransp			+			
	DenTranspRp						+
	DenTranspStCrs						+
	PctAg	+	+			+	+
	PctAgRp				+		
	Pctlmp	+					
	PctUrb		+				
	PctUrbRp				+	+	+
	SlopeTranspStCrs				+		

Key (an asterisk, ‘*’, denotes stressors identified by the Mat-Su Basin Salmon Habitat Partnership):

DenCDP - Density of Canals, Ditches, and Pipelines

DenCulv - *Density of Culverts

DenHouseRp - Density of Housing within the Riparian Zone

DenMat - *Density of Conditional Use Permits for Gravel, *Parcels with Material Sales, *Timber Sales

DenMine - Density of Mines, *Historical Mining Disturbance, *State Prospecting Sites

DenPoll - Density of Contaminated Sites, Wastewater Treatment Facilities, Industrial Facilities

DenResvr - Density of Reservoirs

DenSepSew - *Density of Septic and Sewer

DenSubd - *Density of Cadastral Subdivisions

DenTransp - Density of Transportation (*Airports, Roads, Railroads, Trails)

DenTranspRp - Density of Transportation (*Airports, Railroads, Roads, Trails) in the Riparian Zones

DenTranspStCrs - Density of Transportation (Railroad, Road, Trail)-Stream Intersections

PctAg - Percent of Agricultural Land Cover

PctAgRp - Percent of Agricultural Land Cover in the Riparian Zone

PctImp - Percent Imperviousness of Human-Related Landscapes

PctUrb - Percent of Urban Land Cover

PctUrbRp - Percent of Urban Land Cover in the Riparian Zone

SlopeTranspStCrs - Denssity of Transportation (Railroad, Road, Trail)-Stream Intersections Weighted by the Slope of the Stream Reach
