# Supplementary material for: Applying the index of watershed integrity to the Matanuska-Susitna basin
Source: Arct Antarct Alp Res. Author manuscript; Available in PMC 2021 Aug 27. (PMC7592703; doi:10.1080/15230430.2020.1800219)
Supplement: Supplement1 [file NIHMS1635627-supplement-Supplement1.docx]

Table S1. Stressor name; Units; Use of the stressor in the conterminous United States analysis (0 = no; 1 = yes); Method of Calculation; Data Source; Date of data download (yyy/mm/dd).

| **Stressor** | | **Units** | **Use** | **Method of Calculation** | **Data Source** | **Date** |
| --- | --- | --- | --- | --- | --- | --- |
| DenCDP | | km / km^2^ | - | *Catchment:* V_c_=∑(x_a+_ x_b_)/A_c_, where x_a_ is the total length of canal, ditch or pipeline(s) a and x_b_ is the length of canal, ditch or pipeline(s) b in catchment c, and A_c_ is the area of catchment c.  *Watershed:* V*_w_=*∑(x_a+_ x_b_)/∑A_c_, where x_a_ is the total length of canal, ditch, and pipline(s) a and x_b_ is the total length of canal, ditch, and pipeline(s) b within watershed c, and A_c_ is the area of watershed c for all contributing catchments. | - | - |
| a | Canals, ditches, and pipelines | km | 1 | - | NHDFlowline (attribute: Canal / ditch, pipeline) at <http://akhydro.uaa.alaska.edu/data/nhdplus/> | 2017 / 10 / 10 |
| b | Canals, ditches, and pipelines | km | 1 | - | Personal communication with the U.S. EPA | 2018 / 03 / 16 |
| DenCulv | | culverts / km^2^ | 0 | *Catchment:* V_c_=∑x_i_/A_c_, where x_i_ is the total number of culvert(s) i in catchment c, and A_c_ is the area of catchment c.  *Watershed:* V*_w_=*∑x_c_/∑A_c_, where x_c_ is the total number of culverts within watershed c, and A_c_ is the area of watershed c for all contributing catchments. | Fish Passage Inventory Database at http://www.adfg.alaska.gov/index.cfm?adfg=fishpassage.database | 2018 / 02 / 20 |
| DenHouseRp | | m^2^ / km^2^ | 1 | *Catchment:* V_c_=∑x_i_/A_c_, where x_i_ is the total number of infrastructure i in catchment c, and A_c_ is the area of the riparian zone in catchment c.  *Watershed:* V*_w_=*∑x_c_/∑A_c_, where x_c_ is the total area of infrastructure within watershed c, and A_c_ is the area of the riparian zone in watershed c for all contributing catchments. | Infrastructure Buildings at https://data1-msb.opendata.arcgis.com/datasets/infrastructure-buildings | 2017 / 10 /13 |
| DenMat | | permits or sales / km^2^ | - | *Catchment:* V_c_=∑(x_a+_ x_b+_ x_c_)/A_c_, where x_a_ is the total number of gravel and condition use permit(s) a and x_b_ is the presence of parcel with material sale(s) b and x_c_ is presence of timber sale(s) c in catchment c, and A_c_ is the area of catchment c.  *Watershed:* V*_w_=*∑(x_a+_ x_b+_ x_c_)/∑A_c_, where x_a_ is the total number of gravel and condition use permit(s) a and x_b_ is the presence of parcel with material sale(s) b and x_c_ is presence of timber sale(s) c in watershed c, and A_c_ is the area of watershed c for all contributing catchments. | - | - |
| a | Gravel and Condition Use Permits | permits | 0 | - | Personal communication with the Matanuska-Susitna Borough | 2017 / 12 / 05 |
| b | Parcels with Material (sand or Gravel) Sales | sales | 0 | - | Resource sale at <http://asgdc.alaska.gov/#151> | 2017 / 12 / 05 |
| c | Timber sales | sales | 0 | - | Timber sales at http://forestrymaps.alaska.gov/arcgis/rest/services/Resources/Timbersales/FeatureServer | 2018 / 01 / 12 |
| DenMine | | sites or mines / km^2^ | - | *Catchment:* V_c_=∑(x_a+_ x_b+_ x_c_)/A_c_, where x_a_ is the total number of State Prospecting site(s) a and x_b_ is the presence of historical mining disturbance(s) b and x_c_ is presence of mine(s) c in catchment c, and A_c_ is the area of catchment c.  *Watershed:* V*_w_=*∑(x_a+_ x_b+_ x_c_)/∑A_c_, where x_a_ is the total number of State Prospecting site(s) a and x_b_ is the presence of historical mining disturbance(s) b and x_c_ is presence of mine(s) c in watershed c, and A_c_ is the area of watershed c for all contributing catchments. | - | - |
| a | State (AK) Prospecting Sites | sites | 0 | - | State Prospecting Site at http://www.asgdc.state.ak.us/#138 | 2017 / 12 / 05 |
| b | Historical Mining Disturbance | mines | 0 | - | Historic Mining Footprint at <https://accscatalog.uaa.alaska.edu/dataset/historic-mining-footprint> | 2018 / 01 / 19 |
| c | Mines | mines | 1 | - | Alaska Resource Data File at https://mrdata.usgs.gov/ardf/ | 2017 / 10 / 06 |
| DenPoll | | sites / km^2^ | - | *Catchment:* V_c_=∑(x_a+_ x_b+_ x_c +_ x_d_)/A_c_, where x_a_ is the total number of NPDES site(s) a and x_b_ is the presence of TRI site(s) b and x_c_ is presence of DEC contaminated site(s) c and x_d_ is the presence of a superfund site(s) in catchment c, and A_c_ is the area of catchment c.  *Watershed:* V*_w_=*∑(x_a+_ x_b+_ x_c+_ x_d_)/∑A_c_, where x_a_ is the total number of NPDES site(s) a and x_b_ is the presence of TRI site(s) b and x_c_ is presence of DEC contaminated site(s) c and x_d_ is the presence of a superfund site(s) in watershed c, and A_c_ is the area of watershed c for all contributing catchments. | - | - |
| a | National Pollutant Discharge Elimination System (NPDES) | NPDES sites | 1 | - | National Pollutant Discharge Elimination System at https://echo.epa.gov/tools/data-downloads | 2017 / 10 / 06 |
| b | Toxic Release Inventory (TRI) Sites | TRI sites | 1 | - | Toxic Release Inventory Sites https://www3.epa.gov/enviro/html/fii/downloads/state_files/state_single_ak.zip | 2017 / 10 / 07 |
| c | DEC Contaminated Sites | contaminated sites | 0 | - | Alaska DEC Contaminated Sites at http://www.arcgis.com/home/webmap/viewer.html?webmap=315240bfbaf84aa0b8272ad1cef3cad3 | 2017 / 12 / 14 |
| d | Superfund Sites | superfund sites | 1 | - | Superfund Sites at https://www.epa.gov/superfund/search-superfund-sites-where-you-live | 2017 / 12 / 15 |
| DenResvr | | dams / km^2^ | 1 | *Catchment:* V_c_=∑x_i_/A_c_, where x_i_ is the total number of dam(s) i in catchment c, and A_c_ is the area of catchment c.  *Watershed:* V*_w_=*∑x_c_/∑A_c_, where x_c_ is the total number of dams within watershed c, and A_c_ is the area of watershed c for all contributing catchments. | Dam at http://geonames.usgs.gov/pls/gnispublic/f?p=gnispq:2:::NO::P1_CLASS,P1_STATE:Dam,Alaska | 2017 / 10 / 06 |
| DenSepSew | | cadastres with septic or sewer / km^2^ | - | *Catchment:* V_c_=∑(x_a+_ x_b_)/A_c_, where x_a_ is the total number of cadastres with on-site septic and x_b_ is the total number of cadastres with sewerline in catchment c, and A_c_ is the area of catchment c.  *Watershed:* V*_w_=*∑x_c_/∑A_c_, where x_a_ is the total number of cadastres with on-site septic and x_b_ is the total number of cadastres with sewerline in watershed c, and A_c_ is the area of watershed c for all contributing catchments. | - | - |
| a | On-site Septic | - | 0 | - | Personal communication with Matanuska Susitna Borough | 2017 / 12 / 22 |
| b | Sewerlines | - | 0 | - | Personal communication with Matanuska Susitna Borough | 2017 / 12 / 15 |
| DenSubd | | km of cadastral subdivisions/ km^2^ | 0 | *Catchment:* V_c_=∑x_i_/A_c_, where x_i_ is the total perimeter of cadastral subdivision(s) i in catchment c, and A_c_ is the area of catchment c.  *Watershed:* V*_w_=*∑x_c_/∑A_c_, where x_c_ is total perimeter of cadastral subdivision(s) within watershed c, and A_c_ is the area of watershed c for all contributing catchments. | Cadastral Subdivisions at https://data1-msb.opendata.arcgis.com/datasets/cadastral-subdivisions?geometry=-161.507%2C60.383%2C-137.688%2C63.968 | 2018 / 01 / 11 |
| DenTransp | | km / km^2^ | - | *Catchment:* V_c_=∑(x_a+_ x_b+_ x_c +_ x_d+_ x_e_)/A_c_, where x_a_ is the total length of road(s) a and x_b_ is the length of trail(s) b and x_c_ is length of trail(s) c and x_d_ is the length of railroad(s) d and x_e_ is the length of airstrip(s) e in catchment c, and A_c_ is the area of catchment c.  *Watershed:* V*_w_=*∑(x_a+_ x_b+_ x_c+_ x_d+_ x_e_)/∑A_c_, where x_a_ is the total length of road(s) a and x_b_ is the length of trail(s) b and x_c_ is length of trail(s) c and x_d_ is the length of railroad(s) d and x_e_ is the length of airstrip(s) e in watershed c, and A_c_ is the area of watershed c for all contributing catchments. | - | - |
| a | Roads | km | 1 | - | Roads at http://download.geofabrik.de/north-america.html | 2018 / 01 / 08 |
| b | Trails | km | 0 | - | State Park Trails through ILMA at http://www.asgdc.state.ak.us/#2 | 2018 / 01 / 08 |
| c | Trails | km | 0 | - | RS2477 Trails at http://www.asgdc.state.ak.us/#33 | 2018 / 01 / 08 |
| d | Railroads | km | 0 | - | Railroads 63,360 at http://www.asgdc.state.ak.us/#180 | 2018 / 01 / 08 |
| e | Airstrips | km | 0 | - | Airstrips at http://download.geofabrik.de/north-america.html | 2018 / 01 / 08 |
| DenTranspRp | | km / km^2^ | - | *Catchment:* V_c_=∑(x_a+_ x_b+_ x_c +_ x_d+_ x_e_)/A_c_, where x_a_ is the total length of road(s) in the riparian zone a and x_b_ is the length of trail(s) in the riparian zone b and x_c_ is length of trail(s) in the riparian zone c and x_d_ is the length of railroad(s) in the riparian zone d and x_e_ is the length of airstrip(s) in the riparian zone e in catchment c, and A_c_ is the area of the riparian zone of catchment c.  *Watershed:* V*_w_=*∑(x_a+_ x_b+_ x_c+_ x_d+_ x_e_)/∑A_c_, where x_a_ is the total length of road(s) in the riparian zone a and x_b_ is the length of trail(s) in the riparian zone b and x_c_ is length of trail(s) in the riparian zone c and x_d_ is the length of railroad(s) in the riparian zone d and x_e_ is the length of airstrip(s) in the riparian zone e in watershed c, and A_c_ is the area of the riparian zone in watershed c for all contributing catchments. | - | - |
| a | Roads | km | 1 | - | Roads at http://download.geofabrik.de/north-america.html | 2018 / 01 / 08 |
| b | Trails | km | 0 | - | State Park Trails through ILMA at http://www.asgdc.state.ak.us/#2 | 2018 / 01 / 08 |
| c | Trails | km | 0 | - | RS2477 Trails at http://www.asgdc.state.ak.us/#33 | 2018 / 01 / 08 |
| d | Railroads | km | 0 | - | Railroads 63,360 at http://www.asgdc.state.ak.us/#180 | 2018 / 01 / 08 |
| e | Airstrips | km | 0 | - | Airstrips at http://download.geofabrik.de/north-america.html | 2018 / 01 / 08 |
| DenTranspStCrs | | stream crossings / km^2^ | - | *Catchment:* V_c_=∑(x_a+_ x_b+_ x_c +_ x_d_)/A_c_, where x_a_ is the total number of road(s) a and x_b_ is the number of trail(s) b and x_c_ is number of trail(s) c and x_d_ is the number of railroad(s) d with stream reach(es) e in catchment c, and A_c_ is the area of catchment c.  *Watershed:* V*_w_=*∑(x_a+_ x_b+_ x_c+_ x_d_)/∑A_c_, where x_a_ is the total number of road(s) a and x_b_ is the number of trail(s) b and x_c_ is number of trail(s) c and x_d_ is the number of railroad(s) d with stream reach(es) e in watershed c, and A_c_ is the area of watershed c for all contributing catchments. | - | - |
| a | Roads | - | 1 | - | Roads at http://download.geofabrik.de/north-america.html | 2018 / 01 / 08 |
| b | Trails | - | 0 | - | State Park Trails through ILMA at http://www.asgdc.state.ak.us/#2 | 2018 / 01 / 08 |
| c | Trails | - | 0 | - | RS2477 Trails at http://www.asgdc.state.ak.us/#33 | 2018 / 01 / 08 |
| d | Railroads | - | 0 | - | Railroads 63,360 at http://www.asgdc.state.ak.us/#180 | 2018 / 01 / 08 |
| e | Stream reach | - | 1 | - | Streamline at http://akhydro.uaa.alaska.edu/data/nhdplus/ | 2017 / 10 / 20 |
| PctAg | | % | 1 | *Catchment:* V_c_=∑x_i_/A_c_, where x_i_ is the total agricultural land cover area i in catchment c, and A_c_ is the area of catchment c.  *Watershed:* V*_w_=*∑x_c_/∑A_c_, where x_c_ is total agricultural land cover area within watershed c, and A_c_ is the area of watershed c for all contributing catchments. | 81 Pasture/Hay and 82 Cultivated Crops at <https://www.mrlc.gov/data/nlcd-2011-land-cover-alaska-0> | 2017 / 12 / 06 |
| PctAgRp | | % | 1 | *Catchment:* V_c_=∑x_i_/A_c_, where x_i_ is the total agricultural land cover area i in catchment c, and A_c_ is the area of the riparian zone in catchment c.  *Watershed:* V*_w_=*∑x_c_/∑A_c_, where x_c_ is total agricultural land cover area within watershed c, and A_c_ is the area of the riparian zone in watershed c for all contributing catchments. | 81 Pasture/Hay and 82 Cultivated Crops at <https://www.mrlc.gov/data/nlcd-2011-land-cover-alaska-0> | 2017 / 12 / 06 |
| PctImp | | % | - | *Catchment*: P_c_=100×∑x_i,c_/A_c_, where x_i,c_ is equal to the Advanced Land Observation Satellite impervious surface (x_i,a,c_), where available, OR the Landsat-based NLCD impervious surface (x_i,l,c_), otherwise, for catchment c, and A_c_ is the area of catchment c.  *Watershed:* P_w_=100×∑x_i,c_/A_c_, where x_i,c_ is equal to the Advanced Land Observation Satellite impervious surface (x_i,a,c_), where available, OR the Landsat-based NLCD impervious surface (x_i,l,c_), otherwise, for watershed c, and A_c_ is the area of watershed c. | - | - |
| a | LandSat derived Imperviousness | % | 1 | - | NLCD 2011 Percent Developed Imperviousness Zone 8 at <https://www.mrlc.gov/data/nlcd-2011-percent-developed-imperviousness-zone-8-alaska-0> | 2017 / 12 / 06 |
| b | Advanced Land Observation Satellite derived imperviousness | % | 0 | - | Personal communication with TNC | 2018 / 01 / 11 |
| PctUrb | | % | 1 | *Catchment:* V_c_=∑x_i_/A_c_, where x_i_ is the total urban land cover area i in catchment c, and A_c_ is the area of catchment c.  *Watershed:* V*_w_=*∑x_c_/∑A_c_, where x_c_ is total urban land cover area within watershed c, and A_c_ is the area of watershed c for all contributing catchments. | [21](https://www.mrlc.gov/nlcd11_data.php) Developed, Open Space; 22 Developed, Low Intensity; 23 Developed, Medium Intensity; 24 Developed High Intensity at <https://www.mrlc.gov/data/nlcd-2011-land-cover-alaska-0> | 2017 / 12 / 06 |
| PctUrbRp | | % | 1 | *Catchment:* V_c_=∑x_i_/A_c_, where x_i_ is the total urban land cover area i in catchment c, and A_c_ is the area of the riparian zone in catchment c.  *Watershed:* V*_w_=*∑x_c_/∑A_c_, where x_c_ is total urban land cover area within watershed c, and A_c_ is the area of the riparian zone in watershed c for all contributing catchments. | [21](https://www.mrlc.gov/nlcd11_data.php) Developed, Open Space; 22 Developed, Low Intensity; 23 Developed, Medium Intensity; 24 Developed High Intensity at <https://www.mrlc.gov/data/nlcd-2011-land-cover-alaska-0> | 2017 / 12 / 06 |
| SlopeTranspStCrs | | stream crossings (weighted by slope) / km^2^ | - | *Catchment:* V_c_=∑((x_a x_ x_e_)+(x_b x_ x_e_)+(x_c x_ x_e_)+(x_d x_ x_e_)/A_c_, where x_a_ is road a and x_b_ is trail b and x_c_ is trail c and x_d_ is the railroad d and x_e_ is the slope e in catchment c, and A_c_ is the area of catchment c.  *Watershed:* V*_w_=*∑((x_a+_ x_b+_ x_c_)x_d_)/∑A_c_, where x_a_ is road a and x_b_ is trail b and x_c_ is trail c and x_d_ is the railroad d and x_e_ is the slope e in watershed c, and A_c_ is the area of watershed c for all contributing catchments. | - | - |
| a | Roads | - | 1 | - | Roads at http://download.geofabrik.de/north-america.html | 2018 / 01 / 08 |
| b | Trails | - | 0 | - | State Park Trails through ILMA at http://www.asgdc.state.ak.us/#2 | 2018 / 01 / 08 |
| c | Trails | - | 0 | - | RS2477 Trails at http://www.asgdc.state.ak.us/#33 | 2018 / 01 / 08 |
| d | Railroads | - | 0 | - | Railroads 63,360 at http://www.asgdc.state.ak.us/#180 | 2018 / 01 / 08 |
| e | Slope of stream reach | - | 1 | - | Streamline at http://akhydro.uaa.alaska.edu/data/nhdplus/ | 2017 / 10 / 20 |

Key (an asterisk, ‘*’, denotes stressors identified by the Partnership):

DenCDP - Density of Canals, Ditches, and Pipelines

DenCulv - *Density of Culverts

DenHouseRp - Density of Housing within the Riparian Zone

DenMat - *Density of Conditional Use Permits for Gravel, *Parcels with Material Sales, *Timber Sales

DenMine - Density of Mines, *Historical Mining Disturbance, *State Prospecting Sites

DenPoll - Density of Contaminated Sites, Wastewater Treatment Facilities, Industrial Facilities

DenResvr - Density of Reservoirs

DenSepSew - *Density of Septic and Sewer

DenSubd - *Density of Cadastral Subdivisions

DenTransp - Density of Transportation (*Airports, Roads, Railroads, Trails)

DenTranspRp - Density of Transportation (*Airports, Railroads, Roads, Trails) in the Riparian Zones

DenTranspStCrs - Density of Transportation (Railroad, Road, Trail)-Stream Intersections

PctAg - Percent of Agricultural Land Cover

PctAgRp - Percent of Agricultural Land Cover in the Riparian Zone

PctImp - Percent Imperviousness of Human-Related Landscapes

PctUrb - Percent of Urban Land Cover

PctUrbRp - Percent of Urban Land Cover in the Riparian Zone

SlopeTranspStCrs - Density of Transportation (Railroad, Road, Trail)-Stream Intersections Weighted by the Slope of the Stream Reach
