# Supplementary material for: Applying the index of watershed integrity to the Matanuska-Susitna basin
Source: Arct Antarct Alp Res. Author manuscript; Available in PMC 2021 Aug 27. (PMC7592703; doi:10.1080/15230430.2020.1800219)
Supplement: Supplement2 [file NIHMS1635627-supplement-Supplement2.docx]

Table S2. Stressor, units, total units, total number of catchments, mean catchment value, and maximum catchment value. These values were used to calculate the Index of Catchment integrity and the Index of Watershed Integrity for the Matanuska-Susitna Basin

| **Stressor** | **Units** | **Count (Units)** | **Count (Catchments)** | **Mean Catchment Value** | **Maximum Catchment value (**$\boldsymbol{s}_{\boldsymbol{j,cmax}}$**)** |
| --- | --- | --- | --- | --- | --- |
| DenCDP | km | 2.83 | 6 | 0.47 (SD = 0.46) | 1.43 |
| DenCulv | culverts | 558 | 430 | 1.30 (SD = 0.85) | 10 |
| DenHouseRp | m^2^ | 1131930.96 | 1380 | 820.83 (SD = 2475.49) | 48114.96 |
| DenMat | material sales | 416 | 2382 | 2.02 (SD = 3.69) | 115 |
| DenMine | mine sites | 588 | 544 | 1.08 (SD = 0.42) | 7 |
| DenPoll | sites | 882 | 281 | 3.14 (SD = 8.66) | 129 |
| DenResvr | dams | 7 | 7 | 1 (SD = 0) | 1 |
| DenSepSew | cadasters with sewer or septic | 32413 | 1222 | 26.52 (SD = 130.87) | 3453 |
| DenSubd | perimeter in km | 22735 | 4942 | 4.60 (SD = 11.90) | 436.16 |
| DenTransp | km | 10734.42 | 9298 | 1.15 (SD = 3.62) | 171.64 |
| DenTranspRp | km | 2243.23 | 7359 | 0.30 (SD = 0.45) | 10.89 |
| DenTranspStCrs | crossings | 5194 | 3922 | 1.32 (SD = 0.72) | 14 |
| PctAg | km^2^ | 80.94 | 603 | 0.13 (SD = 0.57) | 8.25 |
| PctAgRp | km^2^ | 4.10 | 279 | 0.01 (SD = 0.03) | 0.24 |
| PctImp | % | N/A | 2293 | 2.55 (SD = 5.36) | 76 |
| PctUrb | km^2^ | 272.17 | 1953 | 0.14 (SD = 0.75) | 14.62 |
| PctUrbRp | km^2^ | 10.60 | 1068 | 0.01 (SD = 0.02) | 0.36 |
| SlopeTranspStCrs | average slope of stream crossing weighted by number of crossings | 629.81 | 3922 | 0.16 (SD = 0.22) | 1.73 |

Key (an asterisk, ‘*’, denotes stressors identified by the Partnership):

DenCDP - Density of Canals, Ditches, and Pipelines

DenCulv - *Density of Culverts

DenHouseRp - Density of Housing within the Riparian Zone

DenMat - *Density of Conditional Use Permits for Gravel, *Parcels with Material Sales, *Timber Sales

DenMine - Density of Mines, *Historical Mining Disturbance, *State Prospecting Sites

DenPoll - Density of Contaminated Sites, Wastewater Treatment Facilities, Industrial Facilities

DenResvr - Density of Reservoirs

DenSepSew - *Density of Septic and Sewer

DenSubd - *Density of Cadastral Subdivisions

DenTransp - Density of Transportation (*Airports, Roads, Railroads, Trails)

DenTranspRp - Density of Transportation (*Airports, Railroads, Roads, Trails) in the Riparian Zones

DenTranspStCrs - Density of Transportation (Railroad, Road, Trail)-Stream Intersections

PctAg - Percent of Agricultural Land Cover

PctAgRp - Percent of Agricultural Land Cover in the Riparian Zone

PctImp - Percent Imperviousness of Human-Related Landscapes

PctUrb - Percent of Urban Land Cover

PctUrbRp - Percent of Urban Land Cover in the Riparian Zone

SlopeTranspStCrs - Density of Transportation (Railroad, Road, Trail)-Stream Intersections Weighted by the Slope of the Stream Reach
